# Analysis of the correlative factors in the selection of interbody fusion cage height in transforaminal lumbar interbody fusion

**DOI:** 10.1186/s12891-016-0866-5

**Published:** 2016-01-12

**Authors:** Hongli Wang, Wenjie Chen, Jianyuan Jiang, Feizhou Lu, Xiaosheng Ma, Xinlei Xia

**Affiliations:** Department of Orthopaedics, Huashan Hospital, Fudan University, Shanghai, 200040 China

**Keywords:** Lumbar spine, Lumbar degenerative disease, Lumbar fusion, Interbody fusion, Interbody cage, Radiologic measurement

## Abstract

**Background:**

Selecting an interbody cage with appropriate height is one of the key steps in lumbar interbody fusion, and has an important impact on clinical efficacy. How to choose the appropriate height of the cage becomes one of the core problems of lumbar interbody fusion for spine surgeons. However, studies about objective selection criteria on interbody cage height was rare.

**Methods:**

One hundred fifty-seven patients with single segment lumbar degenerative diseases treated by TLIF surgery from January 2011 to July 2013 were retrospectively analyzed. Parameters analyzed included: gender, age, body height, clinical diagnosis, pathological segment location and the intervertebral height of pathological segment, pathological segment activity, the intervertebral height of the adjacent segments. And further to analyze the correlation between these parameters and interbody cage height. By measuring the intervertebral height of pathological segment and normal segment to calculate the regression equation of interbody cage height.

**Results:**

The average interbody cage height of male patients (12.38 ± 1.43) mm was significantly higher than female (11.62 ± 1.45) mm (*p* < 0.001). The L4-5 segment interbody cage height (12.11 ± 1.38) mm was significantly greater than the L5-S1 (11.25 ± 1.32) mm (*p* = 0.04). Body height, the intervertebral height of pathological segment, and the middle intervertebral heigh of upper adjacent segment were highly positively correlated to the interbody cage height. The range of interbody cage height used in transforaminal lumbar interbody fusion for Chinese patients with lumbar degenerative diseases was: L3-4 (11.28 ± 3.29) mm ~ (12.76 ± 2.40) mm, L4-5 (11.62 ± 2.89) mm ~ (13.18 ± 1.91) mm, L5-S1 (10.52 ± 2.22) mm ~ (11.90 ± 2.80) mm. The regression equation of interbody cage height was: interbody cage height = 11.123-0.563 * (gender) + 0.149 * (the middle intervertebral height of pathological segment).

**Conclusions:**

The selection of interbody cage height was influenced by sex, body height, pathological segment location, the intervertebral height of pathological segment and other factors. The interbody cage height for the lower lumbar spine mostly selected 11,12,13 mm, L3-4, L4-5 segment highly selective in general should not be less than 10 mm, and L5-S1 segments height was relatively small, usually not more than 13 mm. The interbody cage height might be selected based on the regression equation of interbody cage height. But, the regression equation maybe need to be verified in a prospective study.

## Background

Lumbar fusion has been proved excellent clinical effect after more than 100-year development since Albee FH [1] and Hibbs RA [2] first used in 1911, and has become one of the most commonly used operative methods for treating lumbar degenerative diseases which includes posterolateral fusion and interbody fusions [3–5]. The reconstruction of the sagittal balance has become the important purpose of lumbar fusion surgery [6]. Compared with posterolateral fusion, lumbar interbody fusion could restore the supporting role of the anterior column and intervertebral disc height effectively, and provide immediate postoperative stability, which lead to a gradual increase in the proportion of clinical application in recent years [7–10].

Transforaminal lumbar interbody fusion (TLIF) has been widely performed in recent years [11–17], and could effectively restore the intervertebral height, correct lordosis in the pathological lumbar segment and achieve a satisfactory prognosis [8, 9, 18, 19]. The lumbar fusion cage, which has been generally accepted, is an alternative method to autologous bone graft, and is mainly used to fill the gap in height after the discectomy and the removal of the superior and inferior cartilage endplates in the diseased segment. Meanwhile, the lordosis in the diseased segment is corrected through the strong fixation using the screw-rod system in the maintenance of base height via the fusion cage. Hsieh PC et al. [6] reported that the local disc angle became smaller after TLIF, which was considered to be caused by an inappropriate posterior position of the implanted fusion cage. This result indicated that the height and position of the implanted fusion cage pathological the recovery from lumbar lordosis and the postoperative prognosis.

Selecting an interbody cage with appropriate height is one of the key steps in lumbar interbody fusion, and has an important impact on clinical efficacy. An oversized cage will not only lead to over distraction of intervertebral space, which may increase abnormal stress in the adjacent segments resulting in increasing the incidence of degeneration of the adjacent segments, but will increase the chance to injury the nerve roots. Using a fusion cage too low in height, on the other hand, will fail to restore the intervertebral height and lordosis, and may lead to severe complications such as cage migration and fusion failure [20, 21]. Thus, how to choose the appropriate height of the cage becomes one of the core problems of lumbar interbody fusion for spine surgeons. But the interbody cage height in TLIF has long been determined by surgeons mostly based on their operational experience. Hence, it is meaningful to investigate the related factors in the selection of interbody cage height.

The present study attempt to compare the differences in the height of fusion cages used for different disease types or different affected segments based on body height, age, diagnosis, pathological segment location and imaging data collected from patients treated with TLIF. The aim of this study was to measure the intervertebral disc height of normal and pathological segments affected by degenerative diseases, to investigate the factors related to the selection of fusion cage height and to provide reference for height selection of cages in TLIF for patients with lumbar degenerative diseases. This study was conducted with approval from the Ethics Committee of Huashan Hospital, Fudan University. Written informed consent was obtained from all participants before the operation.

## Methods

### Patient selection

We retrospectively analyzed 157 consecutive patients who underwent TLIF for one-segment lumbar degenerative diseases between January 2011 and July 2013 based on following inclusion criteria: 1). The patients were diagnosed as lumbar degenerative diseases (lumbar disc herniation, lumbar spinal stenosis or lumbar degenerative spondylolisthesis) in L3-4, L4-5 or L5-S1; 2). The patients underwent single-level TLIF with one interbody cage implanted; 3). During the follow-up, there were no complications such as fusion failure, cage retropulsion or pseudarthrosis formation in at least 6 months; 4). The upper and lower adjacent lumbar intervertebral discs were Pfirrmann grade ≤ III [22]. This study was conducted with approval from the Ethics Committee of Huashan Hospital, Fudan University. Written informed consent was obtained from all patients.

The exclusion criteria were as follows: 1). The patients were diagnosed as isthmic spondylolisthesis, lumbar scoliosis, multiple lumbar degenerative diseases, lumbar secondary surgery, one-segment lumbar degenerative diseases in L1-2, L2-3, or one-segment lumbar degenerative diseases with lumbar fractures, severe osteoporosis, spinal canal tumor or other space-occupying lesions. 2). The patients who had two cages implanted. 3). The patients lost of follow-up, or less than 6 months, or had complications such as fusion failure, cage retropulsion or pseudarthrosis formation. 4). There were adjacent lumbar intervertebral discs observed between Pfirrmann Grade IV and Grade V [22].

In the selected patients, there were 68 male patients and 89 female patients, with an average age of 58.0 ± 12.5 years and an average body height of 163.5 ± 8.5 cm. There were 64 patients with lumbar disc herniation, 51 patients with lumbar spinal stenosis and 42 patients with spondylolisthesis. The surgical procedures were performed on L3–4 in 9 patients, L4–5 in 125 patients and L5–S1 in 23 patients (Table 1).

**Table 1:** The basic data of 157 patients with one-segment lumbar degenerative diseases

Disc level	Disease type	Male	Female	Average age (y)	Average height (cm)	LDH	LSS	DLS
L3-4	9	3	6	60.4 ± 10.1	164.1 ± 9.4	3	3	3
L4-5	125	54	71	59.9 ± 11.8	163.0 ± 7.9	48	45	32
L5-S1	23	11	12	48.7 ± 13.3	166.4 ± 7.9	23	3	7
Total	157	68	89	58.0 ± 12.5	163.5 ± 8.5	64	51	42

LDH: lumbar disc herniation, LSS: lumbar spinal stenosis, DLS: degenerative lumbar spondylolisthesis

### Study design

**Fig. 1:**
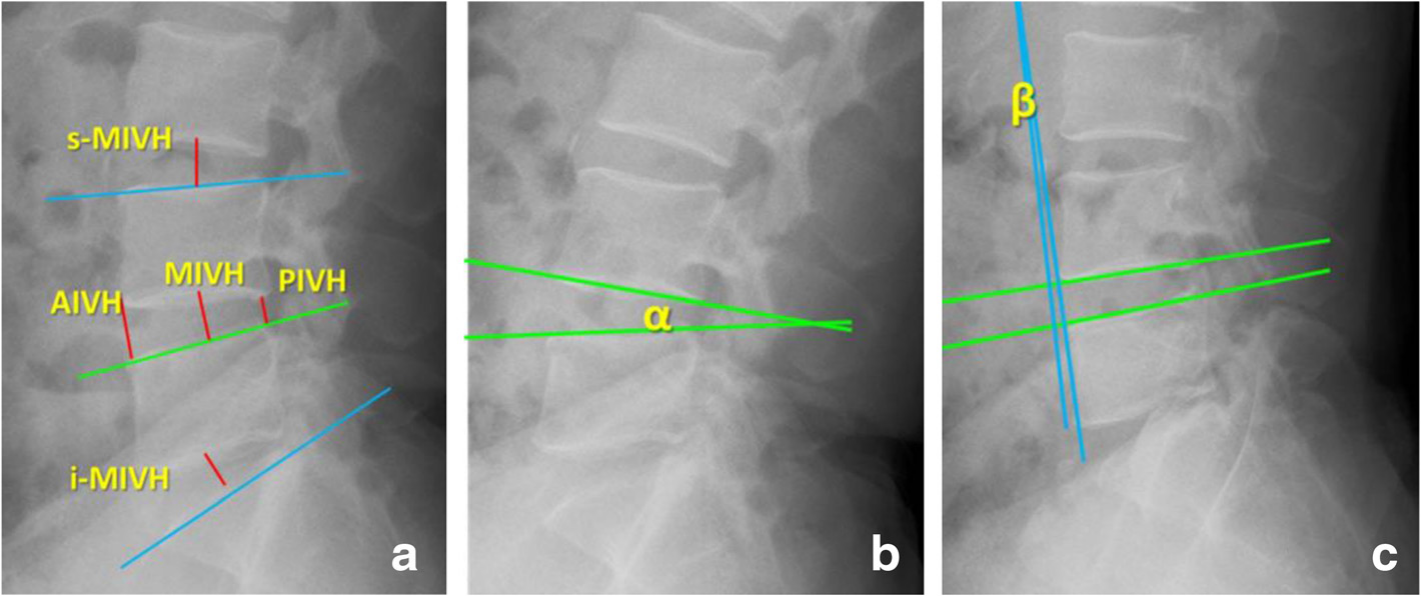
Measurement methods of the intervertebral height and range of motion. **a** Measurement methods of the anterior, posterior intervertebral height (AIVH & PIVH) and the intervertebral height at the midpoint of the pathological segment (MIVH), and the intervertebral height at the midpoint of the superior and inferior segment (s-MIVH and i-MIVH, respectively). **b** and **c** The range of motion (ROM) of the pathological segment is measured based on the Cobb method, and is the difference between Angle α andβ

All enrolled subjects underwent lumbar anteroposterior, lateral and dynamic radiographs. All imaging parameters on the X-ray radiographs were measured by two experienced radiologists using the Centricity Enterprise Web v3.0 (General Electric Com., New York). The following specific measurement parameters were included. 1). The measurement of the intervertebral height taken from the lateral position: One straight line was extended tangent to the inferior endplate of the pathological segment. Three lines were then extended from the anterior, posterior edge and midpoint of the superior endplate vertically toward the line from the inferior endplate, and the length of these vertical line segments were defined as the anterior, posterior intervertebral height (AIVH & PIVH) and the intervertebral height at the midpoint of the pathological segment (MIVH). The same method as described above was used to measure the intervertebral height at the midpoint of the superior and inferior segment, short for s-MIVH and i-MIVH, respectively (Fig. 1a). 2). The range of motion (ROM) of the pathological segment was measured as the difference between the two angles from the radiographs taken at the anterior flexion and posterior extension positions based on the Cobb method (Fig. 1b and c).

### Surgical management

The same group experienced spine surgeons performed all TLIF surgeries operated on each patient using bilateral internal fixation [23]. With adequate intraoperative decompression, a Capstone lumbar interbody fusion cage (Medtronic Sofamor Danek USA Inc., Memphis, Tennessee) was implanted after distraction. The selection criteria of interbody cage height for this group patients as followed: 1) The intervertebral segment had good stability with certain pull-out resistance after implanting the interbody cage. 2) The height and lordosis of operational segment got a good recovery confirmed by intraoperative fluoroscopy. 3) Both the upper and lower bony endplates of operational segment were kept intact,and the lower nerve root was no significant longitudinal traction. Bone fusion in the pathological segment was detected in postoperative follow-up. Among the enrolled subjects, 2 cages at 8 mm, 36 cages at 10 mm, 83 cages at 12 mm and 36 cages at 14 mm were implanted.

### Data analysis

SPSS 19.0 statistical software (SPSS Inc., Chicago, Illinois) was used for the statistical analysis. The difference was statistically significant if *p* < 0.05. Kruskal-Wallis H test was used to compare the differences between the diseases, Wilcoxon test was used to compare the differences between different segments, *t* test was used to compare the differences between MIVH, s-MIVH and s-MIVH, and genders. Associations between the radiological parameters and the interbody cage height were analyzed with Spearman correlation analysis.

## Results

### Differences in the fusion cages used for different disease types

The Kruskal-Wallis H Test showed no differences in the height of the fusion cages used for different disease types (*p* = 0.20). For each disease type, no significant difference was observed in pairwise inter-group comparison (8 mm fusion cages were not included in the analysis due to the small sample size). The average fusion cage height used was 12.16 ± 1.30 mm in the lumbar disc herniation group, 12.00 ± 1.55 mm in the lumbar spinal stenosis group and 11.57 ± 1.43 mm in the lumbar degeneration spondylolisthesis group.

### Differences in fusion cage usage for different segments

Due to the small size of the L3–4 group and the 8 mm fusion cage group, fusion cage usage was only compared between the L4–5 group and the L5–S1 group using the Wilcoxon rank sum test. A p value of 0.04 was obtained, indicating that the average height of the fusion cages used for diseased L4–5 segment (12.11 ± 1.38 mm) was greater than that for the L5–S1 segment (11.25 ± 1.32 mm).

### Differences in fusion cages used between different genders

Significant differences were observed in the average height of the fusion cages used between male and female subjects (*p* < 0.001; the 8 mm fusion cage was not included). The average height of the fusion cages used in male subjects (12.38 ± 1.43 mm) was greater than that used in female subjects (11.62 ± 1.45 mm). (Table 2)

**Table 2:** Statistical data of the selection of fusion cage height with different diagnosis, segments and gender

Fusion cage height	8 mm	10 mm	12 mm	14 mm	Average height (mm)	*p*
LDH	0	11	37	16	12.16 ± 1.30	0.20
LSS	1	12	24	14	12.00 ± 1.55	
DLS	1	13	22	6	11.57 ± 1.43	
L3-4	0	3	2	4	12.22 ± 1.86	0.04
L4-5	1	24	69	31	12.11 ± 1.38	
L5-S1	1	9	12	1	11.25 ± 1.32	
Male	2	6	37	23	12.38 ± 1.43	<0.001
Female	0	30	46	13	11.62 ± 1.45	

LDH: lumbar disc herniation, LSS: lumbar spinal stenosis, DLS: degenerative lumbar spondylolisthesis

### Correlation between fusion cage height and index parameters

A Spearman correlation analysis was performed between the fusion cage height and age, body height, AIVH, MIVH, PIVH, i-MIVH, s-MIVH and ROM of the pathological segment. The fusion cage height showed positive correlations with AIVH, MIVH, PIVH, s-MIVH and body height, with the correlation coefficients in ascending order. With increases in these parameters, the height of the fusion cage increased correspondingly (Table 3).

**Table 3:** Correlation between fusion cage height and index parameters

	Age	Body height	AIVH	MIVH	PIVH	s-MIVH	i-MIVH	ROM
Coefficient of correlation	-	0.169	0.331	0.331	0.314	0.183	-	-
*p*	0.850	0.034	<0.001	<0.001	<0.001	0.022	0.143	0.187

AIVH: anterior posterior intervertebral height, MIVH: intervertebral height at the midpoint, PIVH: posterior intervertebral height, s-MIVH: intervertebral height at the midpoint of the superior segment, i-MIVH: intervertebral height at the midpoint of the inferior segment, ROM: range of motion

### Intervertebral disc height of normal and pathological segments

In the present study, the intervertebral height of 291 segments assessed between Pfirrmann Grade I to Grade III were measured. According to Pfirrmann classification of disc degeneration, the intervertebral disc height of Grade I to Grade III indicate no height loss or slightly decrease [22]. So the adjacent segments could be considered as normal or similar to normal intervertebral disc height. The average MIVH of L2-3 to L5-S1 was 12.15 ± 2.42 mm, 12.76 ± 2.40 mm, 13.18 ± 1.91 mm, 11.90 ± 2.80 mm, respectively (Table 4).

**Table 4:** Intervertebral disc height of normal and pathological segment

Parameters	Number of segments		MIVH		Height of fusion cage
Segments	Pathological segments	Normal segments	Pathological segments	Normal segments	
L3-4	9	125	11.28 ± 3.29	12.76 ± 2.40	12.22 ± 1.86
L4-5	125	32	11.62 ± 2.89	13.18 ± 1.91	12.11 ± 1.38
L5-S1	23	125	10.52 ± 2.22	11.90 ± 2.80	11.25 ± 1.32

MIVH: intervertebral height at the midpoint

### Stepwise regression of fusion cage height

Stepwise regression equations were obtained using the fusion cage height as the dependent variable and the above parameters as the independent variables, which showed significant differences. These variables included gender (male = 1, female = 2), AIVH, MIVH, PIVH, s-MIVH and body height. The stepwise regression equation was significant (*p* < 0.001) with the retained two independent variables, gender and MIVH. The equation was as follows: interbody cage height = 11.123 – 0.563*gender + 0.149*MIVH.

## Discussion

Unlike the prone position the subjects assumed in the fusion surgery, the basic function of the human spine is load-bearing, and thus, the height and curvature formed with the fusion cage implanted in a non-load-bearing state are likely to deviate from those under the normal upright position. Literature [24] reports indicated that the normal human lumbar exhibited significantly increased local disc angle in the L2–3 and L3–4 segments and markedly reduced local disc angle in the L5–S1 segment in response to axial stress. This result suggested that in response to axial stress, the upper lumbar spine tends to disperse the force toward the medial and posterior column, while the lower lumbar spine compresses the intervertebral height to some extent. The differences in the lumbar intervertebral height are determined by the anatomical structure and the physiological characteristics of the lumbar spine itself, which is consistent with the finding from the present study that the height of the fusion cage used in the surgery correlated with the segment pathological by the disease rather than with the disease type itself. On the other hand, Videman T et al. [25] found that the lower lumbar intervertebral height decreased with age in an elderly sample. Meanwhile, large individual variance in the height reduction was found in the same study, which might explain why age was not found to correlate with the height of the fusion cage in the present study.

According to the results from this study, the MIVH of the pathological segments from L3-4 to L5-S1 was 11.28 ± 3.29 mm, 11.62 ± 2.89 mm, 10.52 ± 2.22 mm, which was significantly lower than those of the normal segments: 12.76 ± 2.40 mm, 13.18 ± 1.91 mm and 11.90 ± 2.80 mm, respectively. Therefore, the proper cage height of Chinese population should be within the range of the two sets of data mentioned above of each segment. The average cage height of each segment we actually used was 12.22 ± 1.86 mm, 12.11 ± 1.38 and 11.25 ± 1.32 mm, which, however, was exactly within the proper range that proved the adequacy of the choice of the cage height in this sample. Combined with common cage scale, we recommend that the cage height of L3-4 and L4-5 level via TLIF should be 11, 12 or 13 mm, while for L5-S1 level, 10, 11 or 12 mm would be more appropriate, which means, in Chinese population, the interbody cage height for patients with lumbar degenerative diseases should not be lower than 10 mm in L3-4 and L4-5 segment, and not be greater than 13 mm in L5-S1 segment. In addition, the stepwise regression equation: interbody cage height = 11.123 – 0.563*gender + 0.149*MIVH, presented in the study could be used to estimate the interbody cage height preliminarily during the operation.

There were some limitations in this retrospective study. The small sample size and the large deviation of segmental distribution might have an impact on the accuracy of some parameters, especially for L3-4 segment. The results of L3-4 segment need to be more careful handling. Besides, the selection of the size of the interbody cage for each patient in this sample group was mainly depend on the surgeons’ experience during the operation, of which the correctness should be proved in further studies, despite of the height of the cages used was within the range of the intervertebral height at the midpoint of the pathological and normal segments.

## Conclusion

In summary, the selection of interbody cage height was influenced by gender, body height, pathological segment location, the intervertebral height of pathological segment and other factors. The interbody cage height for the lower lumbar spine mostly selected was 11,12,13 mm, L3-4, L4-5 segment highly selective in general should not be less than 10 mm, and L5-S1 segments height was relatively small, usually not more than 13 mm. The interbody cage height might be calculated before surgery for reference based on the regression equation as follows: interbody cage height = 11.123 – 0.563*gender + 0.149*MIVH. But, the regression equation maybe need to be verified in a prospective study.

## Abbreviations

TLIF: Transforaminal lumbar interbody fusion
LDH: Lumbar disc herniation
LSS: Lumbar spinal stenosis
DLS: Degenerative lumbar spondylolisthesis
AIVH: The anterior intervertebral height
PIVH: The posterior intervertebral height
MIVH: The intervertebral height at the midpoint of the pathological segment
s-MIVH: The intervertebral height at the midpoint of the superior segment
i-MIVH: The intervertebral height at the midpoint of the inferior segment
ROM: The range of motion
